# Caudal epidural steroid injections versus selective nerve root blocks for single-level lumbar spinal stenosis: a study protocol for a randomized controlled trial

**DOI:** 10.1186/s13063-021-05485-1

**Published:** 2021-08-09

**Authors:** Akram Osman, Wei Hu, Jianhua-Sun, Jing Li, Xiao Luo, Nianrong Han, Ehsan Abduhani, Zhenqiang Liu

**Affiliations:** The Second Spine Department of the Fourth School of Clinical Medicine of Xinjiang Medical University, Spine Department of the First Affiliated Hospital of Shihezi University, Shihezi, China

**Keywords:** Caudal epidural steroid injections, Selective nerve root blocks, Lumbar spinal stenosis, Randomized controlled trial

## Abstract

**Background:**

Lumbar spinal stenosis (LSS) is a common degenerative condition associated with old age. Its incidence continues to increase with the rapidly aging population in China. Treatment for LSS usually begins with conservative treatments, as some patients refuse surgical procedures or have surgery contraindications. Caudal epidural steroid injections (CESIs) and selective nerve root blocks (SNRBs) are two commonly used conservative treatments for LSS, which have proven to be effective at relieving LSS symptoms in many studies. However, there are no randomized controlled trials comparing these two procedures. We planned the first study to assess which one of these two procedures is more effective in treating LSS. We will compare the efficacy of these two treatment methods in terms of duration of symptom relief and recurrence rate. We hope our findings will help clinicians choose an optimal treatment for LSS patients.

**Methods/design:**

We plan to conduct a 1-year randomized controlled trial that will include a total of 76 subjects. They will be randomly divided into two groups: group A (patients will receive CESIs) and group B (patients will receive SNRBs). Two days before the procedure, we will assess these patients using the Leeds Assessment of Neuropathic Symptoms and Signs (LANSS) pain scale, Oswestry Disability Index (ODI), and numeric rating scale (NRS) for pain. One day, 2 weeks, 3 months, 6 months, and 1 year after the procedure, we will assess the condition of these patients again with the NRS and ODI.

**Discussion:**

We hope our findings will lay the foundation for the design of further comprehensive studies and help clinicians make a choice between CESIs and SNRBs for LSS patients.

**Trial registration:**

Chinese Clinical Trial Registry ChiCTR1900028038. Registered on 8 December 2019

## Background

Lumbar spinal stenosis (LSS) is a lower back condition where the central canal, lateral recess, or neural foramina becomes narrowed. Common LSS symptoms are lower back pain, burning pain in the buttocks or legs, weakness, numbness or tingling, and loss of sensation [1]. However, some people with LSS may be asymptomatic. Other patients may have to lean forward to relieve pain while walking, but physical examination often fails to find positive signs [2]. LSS is typically caused by degenerative arthritis, and less common causes include space-occupying lesions and fibrosis. The prevalence of LSS is estimated to be 47.2%, and its classical symptoms are neurogenic claudication and lower back pain [3].

Given that the causes of LSS tend to differ among different patients, doctors need to determine how and why a patient develops LSS in order to formulate a treatment plan that works best for him/her. The main pathogenesis of LSS is inflammatory changes around the nerve roots, congestion, and edema, which is also an important cause of root pain and the theoretical basis for corticosteroid injection to improve clinical symptoms [4–6]. Ma et al. believed that long-term inflammation, chronic strain, ligamentum flavum, and facet hypertrophy reduce the normal space available for nerves in the spinal canal and result in direct pressure on nerve roots causing lower back pain [7]. Chagnas et al. pointed out that LSS is secondary to facet joint osteoarthritis, ligamentum flavum hypertrophy, and/or bulging of the intervertebral disc that leads to narrowing of the spaces around neurovascular structures of the spine [8].

To provide a scoring system that can guide LSS treatment and based on the pathogenesis and imaging characteristics of LSS, researchers have proposed the following grading system: grade 0—a normal intervertebral foramen; grade 1—mild foraminal stenosis with perineural fat obliteration in either the transverse or vertical direction; grade 2—moderate foraminal stenosis with perineural fat obliteration in both the transverse and vertical directions; and grade 3—severe foraminal stenosis with nerve root collapse or morphologic change [9, 10].

Most patients seeking treatment for lower back pain have LSS. Treatments for LSS can be conservative and surgical. Conservative management is successful in most patients and is recommended as first-line treatment. Common conservative treatments for LSS include caudal epidural steroid injections (CESIs) and selective nerve root blocks (SNRBs). The reasons for their popularity among primary care physicians are as follows: First, compared with conventional surgical interventions, SNRBs and CESIs greatly reduce the time that physicians are exposed to X-rays, thus protecting their health. Second, SNRBs and CESIs are easy to perform and have a short learning curve.

The efficacy of CESIs has been demonstrated in many studies. Delport et al. analyzed 140 patients treated with epidural steroid injection (ESI) [11]. Fifty-three percent reported improvement in their functional abilities and 74% were at least somewhat satisfied with ESI as a form of treatment. Fukusaki et al. conducted a prospective, randomized, double-masked trial evaluating the efficacy of ESI in 53 patients with LSS [12]. The patients were randomized into three groups: epidural saline injection, epidural local anesthetic, and epidural anesthetic plus steroid. They found that there was a significant difference in the efficacy of the different treatments at 1 month. However, after 3 months, there was no significant difference between the three groups, proving the short-term effect of ESI. Koc et al. reported findings from a prospective, randomized controlled trial of 33 patients comparing the effects of ESI and a conservative inpatient physical therapy program on pain and function for patients with LSS [13]. They found that the ESI group had significant improvement in visual analog scale (VAS) scores at the 2-week follow-up. They concluded that ESI and physical therapy are equally effective in treating LSS up to 6 months of follow-up, while ESI has better short-term efficacy.

A selective nerve root block (SNRB) is a minimally invasive procedure; a needle is inserted into the epidural space in the foramen at the suspected spinal level, and medications such as steroids and local anesthetics are then injected into the area of a specific nerve root [14]. This procedure is considered a very safe and effective treatment option for spinal neuropathy and chronic back pain [15, 16]. Zhang et al. performed SNRBs in 56 LSS patients and found that 80% of them achieved excellent short-term surgery outcomes [17]. Weiner et al. analyzed 30 patients with herniated discs who underwent nerve root blocks and noted immediate relief in 27 patients. During 1–10 years of follow-up, 22 patients (79%) experienced substantial and permanent pain relief [18]. Martin et al. retrospectively analyzed 30 (20 disc herniations, 10 foraminal stenoses) patients with minor sensory/motor deficits who were treated with a SNRB [19]. They found that 87% of patients had rapid and significant regression of pain in 1–4 days, and 60% had permanent resolution of pain. Therefore, they believe that SNRBs are very effective in treating lumbar neuropathy and recommend them as the treatment of choice for this condition.

Narrowing of the spinal canal caused by lumbar spine degeneration usually occurs slowly, over many years or decades. Substantial narrowing compresses the spinal nerves, which causes symptoms including pain at the early stage, and numbness and weakness of the extremities at the middle and late stages, severely affecting patients’ quality of life. Therefore, doctors should aim for early-stage interventions, offer effective treatments in the middle stage, and absolutely avoid late-stage surgery. In addition, it is recommended that patients in need of conservative treatments receive interventions as early as possible to prevent exacerbation of lumbar neuropathy.

It follows from the above that both CESIs and SNRBs can effectively relieve clinical symptoms and improve neurologic function in patients with LSS. SNRBs are different from epidurals. Instead of administering medication to cover all the nerve roots, selective blocks are performed to target a particular pinched nerve root. SNRB injections can therefore be performed to manage a specific area of pathology without causing damage to nearby normal nerve tissues.

To our knowledge, however, there have been no randomized controlled trials comparing the efficacy of SNRBs and CESIs. In clinical practice, primary care physicians often have to choose between a selective nerve block and a caudal epidural injection based on their own experience, as there is no guideline for them to refer to. To fill this gap, we plan to conduct the first study to compare the effects of SNRBs and CESIs on LSS. We will use outcome measures such as duration of symptom relief and recurrence rate to determine whether SNRBs or CESIs are more effective. We hope our findings will help physicians make a choice between SNRBs and CESIs for LSS patients.

### Hypothesis

Our hypothesis is that SNRBs have a lower recurrence rate than CESIs in treating patients with single-level LSS.

## Methods/design

This is a randomized controlled trial, aimed at comparing the recurrence rate of LSS following treatment with SNRBs and CESIs in patients with one-level LSS. This study was approved by the Ethics Committee of the Fourth School of Clinical Medicine of Xinjiang Medical University and will be conducted in accordance with the World Medical Association Declaration of Helsinki. This study is registered in the Chinese Clinical Trial Registry (Registration No. ChiCTR1900028038). Written informed consent will be obtained from all patients before initiating treatment.

**Fig. 1:**
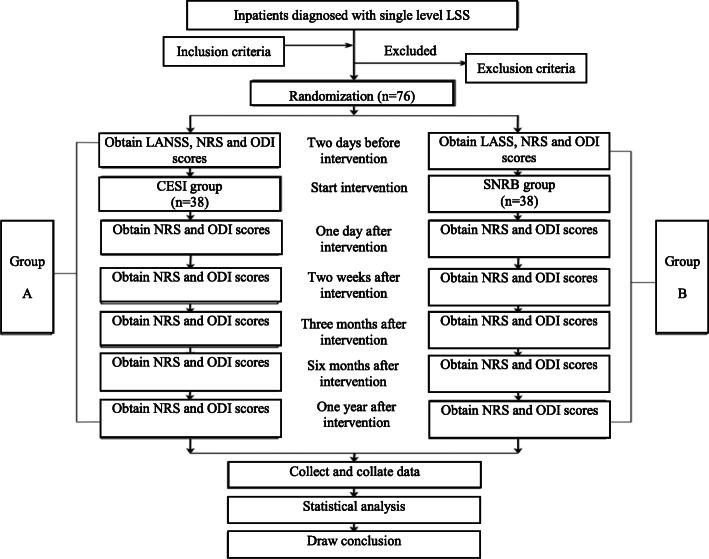
Schematic presentation of patient flow. LSS, lumbar spinal stenosis; CESI, caudal epidural steroid injection; SNRB, selective nerve root block; NRS, numeric rating scale; ODI, Oswestry Disability Index; LANSS, Leeds assessment of neuropathic symptoms and signs

Evaluation of the results and statistical analysis will be performed by professionals who are blinded to the group assignment. Participant flow is shown in Fig. 1. The study will last for 1 year. Patients will be randomly divided into two groups with patients in group A receiving a CESI and patients in group B receiving an SNRB.

The main outcome measures include the Leeds Assessment of Neuropathic Symptoms and Signs (LANSS) pain scale to determine whether the pain experienced is due to nerve damage, the Oswestry Disability Index (ODI) to assess functional status, and a numeric rating scale (NRS) for pain to evaluate pain intensity [20]. Two days before the procedure, we will assess the condition of patients using the LANSS pain scale, ODI, and NRS for pain. One day, 2 weeks, 3 months, 6 months, and 1 year after the procedure, we will evaluate these patients again using the NRS for pain and the ODI.

### Recruitment

First, we will assess the potential candidates and inform them of our study procedure. We will randomly divide the eligible patients into two groups after obtaining written informed consent from each patient. Treatment will be given to each patient based on the group he/she is in.

### Sample size

Because the present study is a clinical application, according to the actual situation, it is difficult to calculate the sample size by pre-experiment, so we finally decided that the sample size of this study was obtained from the previous relevant literature data. The sample size outcome effect size is based on studies found in the literature using the ODI and NRS scores [21]. The mean change of ODI and NRS in group B is expected to be 93% and 0–1 scores, while in group A it is expected to be 80% and 3–4 scores. The sample size was calculated using the sample size formula for a quantitative equivalence trial: *n* = 2(*Z*_*α*_ + *Z*_*β*_)^2^*δ*^2^/(*D* − Δ)^2^ (*α* = 0.05, *β* = 0.2). After we entered *δ* of 2.17, *D* of 0.1, and *Δ* of 1.2 into the formula, we found that it required 31 patients in each group. Given a loss to follow-up rate of 20%, approximately 76 subjects are required.

This experiment was conducted simultaneously in the Second Spine Department of the Fourth School of Clinical Medicine of Xinjiang Medical University and Spine Department of the First Affiliated Hospital of Shihezi University, and inpatients meeting the inclusion criteria were included in the study.

Since the LSS patient base of the above two departments is large enough to recruit subjects at the same time, there is no need to worry about the sample size. Some benefits can also be provided to patients participating in the study to supplement the sample size (such as free professional counseling, functional exercise instruction)

### Inclusion, exclusion, and diagnostic criteria

The inclusion criteria are shown in Table 1. The exclusion criteria are shown in Table 2. All recruited patients who meet the diagnostic criteria for LSS will be screened for eligibility in strict accordance with the inclusion and exclusion criteria.

**Table 1:** Inclusion criteria

1) Lumbar MRI scan shows LSS at a single level (sagittal diameter of lumbar central canal < 13 mm; dural sac area < 100 mm^2^), with no prolapsed or sequestered discs.	
2) Recurrent intermittent claudication and unable to walk long distances.	
3) Aged 50–85 years and in need of pain relief.	
4) Inflammation-induced pain.	
5) Less than a 3-month history of radiating pain in the lower legs.	
6) LANSS score ≥ 12.	

*MRI*, magnetic resonance imaging; *LSS*, lumbar spinal stenosis; *LANSS*, Leeds Assessment of Neuropathic Symptoms and Signs

**Table 2:** Exclusion criteria

1) Patients unable or unwilling to receive treatment	
2) Allergic to contrast medium or drugs planned to be injected	
3) Untreated local infection at the planned surgical site	
4) Patients who cannot cooperate during the procedure or have a history of mental disease	
5) Pregnancy	
6) Receiving anticoagulant therapy	
7) Congenital or surgical anatomical diseases which affect the safety and success of the procedure	
8) Systemic infection	
9) Severe respiratory or cardiovascular diseases	
10) Immunosuppression	

### Diagnostic criteria

According to the “Clinical Guideline for the Diagnosis and Treatment of Degenerative Lumbar Spinal Stenosis by the North American Spine Society (NASS)” in 2011: Degenerative lumbar spinal stenosis describes a condition in which there is diminished space available for the neural and vascular elements in the lumbar spine secondary to degenerative changes in the spinal canal.

The main clinical symptoms include sacroiliac joint pain and sharp radiating pain in the lateral thighs, lower legs, and feet, as well as lower extremity numbness and decreased muscle strength. Some patients also experience intermittent claudication. Leg pain and weakness are precipitated by walking or standing for a long time and relieved with forward flexion, sitting, and rest.

Magnetic resonance imaging (MRI) represents the gold standard for the assessment of LSS: sagittal diameter of the lumbar central canal, 13–15 mm (abnormal), 10–13 mm (narrow), and < 10 mm (absolute stenosis); dural sac area, < 100 mm^2^ (relative stenosis) and < 70 mm^2^ (absolute stenosis).

### Randomization and blinding

Dr. Jianhua Sun and Dr. Jing Li from the Spine Department of the First Affiliated Hospital, School of Medicine, Shihezi University, will use computer software to generate random sequences, which will be sealed in envelopes and concealed from recruiters and subjects. Ehsan from the Second Spine Department and investigator Akram are responsible for recruiting eligible patients. Zhenqiang Liu from the Second Spine Department will randomly assign recruited patients into two groups. In this study, we will use the single-blind approach in which the participant is unaware of which treatment he/she is receiving. The specific procedure performed will be kept hidden from all participants before unblinding.

We also plan to charge all subjects the same fee to ensure the nature of blinding. The specific fees charged are as follows: nerve blocks 86 yuan + 2 vials of triamcinolone acetonide injection (40 mg/ml, Kunming Jida Pharmaceutical Co., Ltd.), 18.98 yuan + 1 vial of lidocaine (20 mg/ml, Hebei Tiancheng Pharmaceutical Co., Ltd.), and 2.62 yuan + 1 vial of Omnipaque (300 mg I/ml, GE Healthcare, Shanghai, China) (83 yuan). The total cost is 190.6 yuan.

### Interventions

Patients will be randomly divided into two groups: group A (patients will receive CESIs) and group B (patients will receive SNRBs). Two days before the procedure, we will assess the condition of patients using the LANSS pain scale, ODI, and NRS for pain.

### Technique for caudal epidural injections

The patient will be asked to lie in the prone position. A soft pillow approximately 10 mm thick is placed under the stomach area so that the sacrum is slightly elevated. The sacral hiatus is identified and marked on the skin. Following correct skin sterilization and draping, a surgeon will first practice inserting a 7-gauge spinal needle. A subjective feeling of “give” and loss of resistance suggests piercing the sacrococcygeal ligament.

An 18-gauge spinal needle is then introduced into the anterior wall of the sacrum. Care is taken to ensure that the needle shaft is at an angle of 90° to the skin surface. Once the needle reaches the periosteum, it is tilted towards the caudal vertebra and advanced upward for up to 5 cm into the sacral canal with an angulation of 30° to the surface. The hub of the needle is then attached to the syringe. Loss of resistance when pushing air is characteristic of entering the sacral canal. After removal of the stylet and guidewire, and aspiration to check for blood or cerebrospinal fluid, 0.2–0.3 ml of Omnipaque is injected to confirm correct placement. If the contrast agent can be seen in the epidural space as confirmed by X-ray imaging, the needle is correctly placed. A mixture consisting of 80 mg of triamcinolone acetonide and 2 ml of 1% lidocaine is injected [22–24].

### Technique for nerve root blocks

The patient is placed in the prone position. The affected level is identified under C-arm guidance, and a horizontal line is marked at the level of the lower edge of the pedicle. After sterile preparation and draping, an 18-gauge spinal needle is inserted 0.5 cm below the marked line and 10 cm from the spinous process at an angle of 25–30° to the coronal plane and parallel to the intervertebral space on the axial plane. For local anesthesia, 1% lidocaine is injected when advancing the needle. Under C-arm guidance, the direction and depth of the needle are observed, and it is inserted where the nerve root exits. Anteroposterior and lateral X-ray imaging of the lumbar spine is performed again when inserting the needle through the intervertebral foramen. On the anterior-posterior view, the tip of the needle is placed approximately 0.5 cm beneath the corresponding pedicle. On the lateral view, the needle tip is positioned just below the pedicle and into 1/3 of the posterior aspect of the intervertebral foramen. Radiating pain along the nerve occurs if the nerve root is punctured. The patient is asked whether this is the site he/she experienced pain. Then, 0.2 to 0.3 ml of Omnipaque is injected into the nerve root sheath to clearly visualize the existing nerve root. When an adequate flow of contrast medium to the target area occurs, a solution consisting of 80 mg of triamcinolone acetonide and 2 ml of 1% lidocaine is injected [25–27].

### Data collection

We will evaluate the treatment outcomes using the NRS and ODI 1 day, 2 weeks, 3 months, 6 months, and 1 year after the procedure. The treatment plan and evaluation of results are shown in Table 3.

**Table 3:** Treatment plan and evaluation of results

Data collection/recording	Person in charge	Observational phase		Follow-up phase			
		2 days before intervention	1 day after intervention	2 weeks after intervention	3 months after intervention	6 months after intervention	1 year after intervention
Obtaining informed consent	Luo Xiao	×					
Collecting patients’ basic information and medical history	Han Nirong	×					
Screen patients for eligibility	Ehsan Abduhani	×					
LANSS pain scale	Akram Osman	×					
ODI	Akram Osman Liu Zhenqiang	×	×	×	×	×	×
NRS for pain	Akram Osman Liu Zhenqiang	×	×	×	×	×	×

*LANSS*, Leeds Assessment of Neuropathic Symptoms and Signs Pain Scale; *ODI*, Oswestry Disability Index; *NRS*, numeric rating scale

### Outcome measures

Multiple outcome measures were utilized which included the NRS (0–10 scale) pain scale and the ODI on a 0–50 scale, with assessment at 1 day, 2 weeks, 3 months, 6 months, and 1 year after the procedure. The Oswestry Disability Index (ODI): back pain-related dysfunction will be assessed using the ODI. The ODI contains 10 questions about daily activities, including inventories of pain intensity, personal care, lifting, walking, sitting, standing, sleeping, sexual life, social life, and traveling. Each question is rated on a scale from 0 to 5 points; the lower the score, the less disabled the person is by the pain. The NRS represented no pain with a 0 and the worst pain imaginable with a 10. NRS is an instrument for the self-assessment of pain and comprises a scale with 0 and 10 at either end of a straight line. The participants marked a point on the line to indicate their level of average pain. The value and validity of the NRS and ODI have been reported [28, 29]. The main outcome measures of the present study were ODI and NRS scores after follow-up and the final recurrence rate calculated according to the requirements. ODI and NRS scores were obtained by designated personnel after follow-up work. After obtaining the above two scoring data, the curative effect was evaluated according to the requirements of Table 4, and the recurrence rate was obtained. According to the ultimate purpose of this study, the recurrence rate plays a very important role in the outcome indicators. ODI and NRS also provide the basis for the final calculation of recurrence rate, which significantly affects the evaluation of the efficacy of each subject, and also plays a decisive role in the research results. Specific criteria for evaluating the efficacy are shown in Table 4.

**Table 4:** Efficacy evaluation

Cured: neither CESI nor SNRB cured LSS	
Marked effect: NRS score < 2 points or ODI score < 50%	
Effective: NRS score 2–4 points or ODI score 50–70%	
Relapse: NRS score > 4 points or ODI score > 70% or the use of analgesics of the patient’s own choice	

*Note*: We will record the time a patient has recurrence and stop follow-up

*CESI*, caudal epidural steroid injection; *SNRB*, selective nerve root block; *LSS*, lumbar spinal stenosis; *NRS*, numeric rating scale; *ODI*, Oswestry Disability Index

### Supervision, safety, and quality control

During the 1-year follow-up period, we will use paper case report forms and self-reported questionnaires to collect data. All data collection will be supervised in strict accordance with the standard procedure of the clinical trial center to ensure full compliance with International Council for Harmonization (ICH) standards and Good Clinical Practice (GCP) principles. Any patient who experiences adverse events such as worsening of clinical symptoms and loss of ability to perform daily activities during the study period will be fully evaluated by doctors’ inquiries, physical examination, and imaging studies. If the adverse event is believed to be caused by the intervention received, the treatment will be terminated and the patient will be offered symptomatic treatment. We will analyze relevant data on adverse events and LSS interventions to improve treatment. All adverse events will also be reported to the investigators. Given the short period and low risk of our study, we did not set up a Trial Steering Committee. Any unexpected events will be directly reported to the investigators for advice. There is no interim analysis was set in this protocol, and WH and AO will have access to decide whether to make the final decision to terminate the trial.

For the control group and the experimental group, other treatments allowed for the patients are as follows: (1) both groups can be given routine nursing treatment in Department II of Spine of Traditional Chinese Medicine Hospital of Xinjiang Uygur Autonomous Region, including daily inquiry of patients’ condition, pain improvement, and care for patients. (2) Acupuncture and moxibustion treatment of lumbar spinal stenosis was performed by a doctor designated by the Acupuncture and Moxibustion Department of the said hospital. Standardized acupuncture and moxibustion treatment of lumbar spinal stenosis was adopted, that is, acupuncture and moxibustion treatment was the same for all subjects. (3) The massage treatment of lumbar spinal stenosis was performed by the designated physician in the Department of Massage of the said hospital. The standardized treatment of lumbar spinal stenosis with acupuncture and massage was used, that is, all subjects had the same massage treatment. (4) The treatment can be performed by the designated nurse of Department II of Spine and ensure that all subjects are electrically treated in the same position. The other treatments allowed are traditional Chinese medicine treatment, which can play the role of adjuvant treatment, and the two groups of patients received the same treatment. As a result, the effect of the above treatment on the experimental results can be avoided. For the control group and experimental group, the other treatments prohibited from the patients are as follows: (1) In the course of the experiment, other invasive treatments for lumbar spinal stenosis were performed, (2) It is forbidden for the subjects to take drugs that can improve the clinical symptoms of lumbar spinal stenosis. (3) Patients were not allowed to receive other treatments for lumbar spinal stenosis without the consent of the researcher. (4) They took related analgesics by themselves.

Xiao Luo, a physician of Spine Department II, had an informed conversation with the subjects 2 days before the operation and obtained consent from the subjects. After Dr. Luo Xiao obtained the informed consent from the subjects, Nianrong Han, a physician of Spine Department II, asked the subjects for the basic information and related medical history of the patients required for the experiment, which was also completed 2 days before the operation. Ehsan Abduhani, a physician of Spine Department II, checked whether the subjects met the prescribed criteria 1 day before the operation and finally determined whether they were included in the experiment. Researcher Akram Osman completed his work of LANSS scoring 2 days before the operation after Dr. Luo Xiao and Dr. Han Nianrong completed their work. After then, Akram Osman and Zhengqing Liu completed the follow-up collection of ODI and NRS. It should be noted that for the same subject, the same person should always follow up and collect data, which cannot be carried out alternately. The follow-up collection of data must be completed within 24 h on the specified date. Akram Osman is responsible for sorting and inputting the collected data into the designated data collection table and setting the password to save independently. This work is completed within 24 h after receiving the data.

### Dropouts and loss to follow-up

All patients have the right to drop out of the study at any time. If a patient chooses to discontinue participation, we will withdraw consent from this patient and terminate the study. The chief investigator can also terminate a subject’s participation if he/she breaks the rules governing this study or has severe adverse events. Data on these subjects will be excluded from our final analysis.

### Statistical methods

Two investigators will use EpiData 3.0 for data entry. SPSS18.0 will be used to analyze all data. All patients’ demographic and clinical characteristics (e.g., gender, age, and weight) will be subject to descriptive analysis. Quantitative data will be expressed as mean ± standard deviation, medians, and ranges, while qualitative data will be expressed as frequency and percentage. A chi-squared test will be used to compare the incidence of adverse events between patients in the two groups.

### Publication of study results

The study results will be published in relevant medical journals. The chief investigator together with the members of the research management team and other investigators will write the manuscript and submit it to a journal for publication.

## Discussion

Both SNRBs and CESIs have proven efficacy against LSS. However, there are no studies comparing the effectiveness of these two treatments in LSS. In clinical practice, primary care physicians often have to choose between these two procedures based on their own experience as there is no guideline for them to refer to. Such subjectivity raises the risk of adopting a suboptimal treatment plan for a patient, which can result in less than desirable outcomes and increase the uncertainty surrounding the prognosis of patients. In severe cases, treatment can fail, increasing patients’ pain and economic burden. To avoid patients receiving inappropriate treatment, we evaluated the effects of SNRB and CESI treatment for LSS. We will divide recruited LSS patients into two groups and offer them either SNRBs or CESIs. Data including duration of symptom relief and time to relapse in patients in the two groups will be recorded, and the recurrence rate will be calculated to determine whether SNRBs or CESIs are more effective in relieving patients’ symptoms long term. We hope our findings will provide a reference for physicians and help them make a choice between SNRBs and CESIs for LSS patients based on scientific and clinical evidence so that these patients can receive optimal treatment with maximal efficacy.

### Trial status

The version number of this protocol is no. 3.0, date: 2020-1-28. The date of subject start recruitment is 2020-4-16, and we expect to complete all research processes by the end of 2021.

## Data Availability

Only AO and HW will have access to the final trial dataset, and the rest are barred from accessing the data. Any data required to support the protocol can be supplied on request.

## Abbreviations

LSS: Lumbar spinal stenosis
LANSS: Leeds Assessment of Neuropathic Symptoms and Signs
ODI: Oswestry Disability Index
NRS: Numeric rating scale
